# Implementation of information and communication technologies for health in Bangladesh

**DOI:** 10.2471/BLT.15.153684

**Published:** 2015-08-31

**Authors:** Sheik Mohammed Shariful Islam, Reshman Tabassum

**Affiliations:** a Center for Control of Chronic Diseases, International Center for Diarrhoeal Diseases Research, Bangladesh (ICDDR,B), 68 Shaheed Tajuddin Ahmed Sarani, Mohakhali, Dhaka 1212, Bangladesh.; b Macquarie University, Sydney, Australia.

## Abstract

**Problem:**

Bangladesh has yet to develop a fully integrated health information system infrastructure that is critical to guiding policy development and planning.

**Approach:**

Initial pilot telemedicine and eHealth programmes were not coordinated at national level. However, in 2011, a national eHealth policy was implemented.

**Local setting:**

Bangladesh has made substantial improvements to its health system. However, the country still faces public health challenges with limited and inequitable access to health services and lack of adequate resources to meet the demands of the population.

**Relevant changes:**

In 2008, eHealth services were introduced, including computerization of health facilities at sub-district levels, internet connections, internet servers and an mHealth service for communicating with health-care providers. Health facilities at sub-district levels were provided with internet connections and servers. In 482 *upazila* health complexes and district hospitals, an mHealth service was set-up where an on-duty doctor is available for patients at all hours to provide consultations by mobile phone. A government operated telemedicine service was initiated and by 2014, 43 fully equipped centres were in service. These centres provide medical consultations by qualified physicians to patients visiting rural and remote community clinics and union health centres.

**Lessons learnt:**

Despite early pilot interventions and successful implementation, progress in adopting eHealth strategies in Bangladesh has been slow. There is a lack of common standards on information technology for health, which causes difficulties in data management and sharing among different databases. Limited internet bandwidth and the high cost of infrastructure and software development are barriers to adoption of these technologies.

## Résumé

**Problème:**

Le Bangladesh n'a pas encore développé d'infrastructure pour mettre en place un système d'information sanitaire totalement intégré, essentiel pour orienter l'élaboration des politiques et la planification.

**Approche:**

Les premiers programmes pilotes de télémédecine et de télésanté n’ont pas été coordonnés à l'échelle nationale. Cependant, en 2011, une politique nationale de télésanté a été mise en œuvre.

**Environnement local:**

Le Bangladesh a apporté des améliorations significatives à son système de santé. Toutefois, le pays rencontre encore des difficultés en matière de santé publique, avec un accès limité et inéquitable aux services de santé et des ressources insuffisantes pour répondre aux demandes de la population.

**Changements significatifs:**

En 2008, des services de télésanté ont été mis en place, notamment l'informatisation des établissements de santé au niveau des sous-districts, des connexions Internet, des serveurs Internet et un service de santé mobile permettant de communiquer avec les professionnels de santé. Dans les sous-districts, les établissements de santé ont été dotés de serveurs et de connexions Internet. Dans 482 centres de santé d'upazilas et hôpitaux de districts, un service de santé mobile a été mis en place : un médecin est à la disposition des patients à toute heure pour réaliser des consultations par téléphone mobile. Un service de télémédecine géré par le gouvernement a été lancé et, en 2014, 43 centres totalement équipés étaient en service. Grâce à ces centres, les patients peuvent se rendre dans des centres de santé ou des cliniques communautaires ruraux et isolés et bénéficier de consultations médicales assurées par des médecins qualifiés.

**Leçons tirées:**

En dépit d'interventions pilotes précoces et d'une mise en œuvre réussie, l'adoption de stratégies de télésanté au Bangladesh progresse lentement. Le pays n'a pas de normes communes en matière de technologies de l'information pour la santé, ce qui entraîne des difficultés dans la gestion et le partage de données entre différentes bases de données. Le débit limité d'Internet ainsi que le coût élevé du développement d'infrastructures et de logiciels freinent l'adoption de ces technologies.

## Resumen

**Situación:**

Bangladesh aún debe desarrollar una infraestructura de sistema de información sanitaria totalmente integrado, cosa que es crucial para tener guías sobre la política de desarrollo y la planificación.

**Enfoque:**

Los programas pilotos iniciales en telemedicina y sanidad electrónica no se coordinaron a nivel nacional. Sin embargo, en 2011 se implementó una política nacional de sanidad electrónica.

**Marco regional:**

Bangladesh ha hecho notables mejoras en su sistema sanitario. Sin embargo, el país sigue afrontando retos en la sanidad pública debido al acceso limitado y desigual a los servicios sanitarios, así como una falta de recursos adecuados para cumplir con las demandas de la población.

**Cambios importantes:**

En 2008, se introdujeron servicios de sanidad electrónica, incluyendo la informatización de los centros sanitarios a nivel de los subdistritos, la conexión y los servidores de Internet y el servicio de sanidad móvil para comunicarse con los profesionales sanitarios. A los centros sanitarios a nivel de los subdistritos se les suministraron servidores y conexión a Internet. En 482 de los complejos sanitarios de las *upazila* y hospitales de distrito se instaló un servicio de sanidad móvil mediante el cual un doctor de servicio está disponible a todas horas para consultas a través de un teléfono móvil. Se inició un servicio de telemedicina operado por el gobierno y en 2014 ya había capacitados 43 centros totalmente equipados. Dichos centros ofrecen consultas médicas hechas por médicos cualificados a pacientes mediante visitas a clínicas comunitarias en zonas rurales y remotas, así como a centros de asistencia médica sindicales.

**Lecciones aprendidas:**

A pesar de las intervenciones piloto iniciales y la exitosa implementación, el progreso de adopción de servicios de sanidad electrónica en Bangladesh ha sido lento. Hay una falta de normas comunes en tecnologías de la información sanitaria, cosa que hace que haya dificultades en la gestión de los datos y en compartirlos entre diferentes bases de datos. El limitado ancho de banda de Internet y los altos costes de la infraestructura y el desarrollo de programas representan barreras para adoptar dichas tecnologías.

## ملخص

**المشكلة:**

لم تتوفر في بنغلاديش حتى الآن البنية التحتية اللازمة لنظام كامل متكامل للمعلومات الصحية والتي تلعب دورًا بالغ الأهمية في وضع سياسة توجيهية والتخطيط لها.

**الأسلوب:**

لم يتم التنسيق لبرامج أولية تجريبية للعلاج عن بُعد أو للصحة الإلكترونية على المستوى الوطني. وعلى الرغم من ذلك، تم تنفيذ سياسة الصحة الإلكترونية على المستوى الوطني في عام 2011.

**المواقع المحلية:**

أجرت بنغلاديش تحسينات كبيرة في نظام الخدمات الصحية لديها. وعلى الرغم من ذلك، ما زالت تواجه هذه الدولة تحديات في مجال الصحة العامة بما يعانيه أهلها من الاستفادة المحدودة وغير العادلة من الخدمات الصحية وغياب الموارد الكافية لتلبية متطلبات السكان هناك.

**التغيرات ذات الصلة:**

تم توفير خدمات الصحة الإلكترونية في عام 2008، بما في ذلك التحول إلى استخدام أجهزة الكمبيوتر بالمنشآت الصحية على مستوى المقاطعات الفرعية، واستخدام طرق الاتصال بالإنترنت، وخوادم الإنترنت، والاستفادة من خدمة التقنيات المتنقلة في مجال الصحة للتواصل مع مقدمي خدمات الرعاية الصحية. وتم تزويد المنشآت الصحية على مستوى المقاطعات الفرعية بسبل الاتصال بالإنترنت والخوادم. وتم إعداد خدمة التقنيات المتنقلة في مجال الصحة في 482 من مجمعات المنشآت الصحية والمستشفيات بالمقاطعات في *يوبازيلا *، حيث يتوفر طبيب مناوب لخدمة المرضى في جميع ساعات اليوم لتقديم الاستشارة الطبية لهم عبر الهاتف المحمول. وقد بدأت الاستفادة من خدمة العلاج عن بُعد التي توفرها الحكومة، وبحلول عام 2014 كان 43 مركزاً مزوداً بالكامل بالأجهزة اللازمة يقدم هذه الخدمة للجمهور. حيث قدمت هذه المراكز الاستشارة الطبية على يد أطباء مؤهلين للمرضى الذين يتوجهون إلى العيادات الطبية المجتمعية والمراكز الصحية التابعة للاتحاد الواقعة في المناطق الريفية والمناطق النائية.

**الدروس المستفادة:**

برغم وجود التدخلات التجريبية في وقت مبكر والنجاح في تنفيذها، ما زالت بنغلاديش تعاني من البطء في التقدم نحو استخدام استراتيجيات الصحة الإلكترونية. فهي تفتقر إلى المعايير العامة التي تنظم استخدام تقنية المعلومات فيما يتعلق بمجال الصحة، مما يسبب صعوبات في إدارة البيانات والمشاركة فيما بين مختلف قواعد البيانات. ويقف المستوى المحدود للنطاق الترددي للإنترنت، وارتفاع تكاليف البنية التحتية وتطوير برامج الكمبيوتر عائقًا في سبيل استخدام هذه التقنيات.

## 摘要

**问题:**

孟加拉国仍未建立起全面完整的医疗卫生信息系统基础设施，而该系统对指导性政策的发展和规划来说非常关键。

**方法:**

初步的试点远程医疗和电子医疗计划在国家层面不协调。 然而，2011 年开始实施了一项国家电子医疗政策。

**当地状况:**

孟加拉国已经对其医疗卫生系统做出了大幅改进。 然而，该国仍然面临公共医疗卫生方面的挑战，包括有限的、不公平的医疗服务使用权，以及缺乏足够的资源来满足人口的需求。

**相关变化:**

2008 年引进了电子医疗服务，包括乡镇级医疗卫生设施电脑化、网络连接、网络服务器和移动医疗服务，方便与医疗服务提供者进行交流。 所提供的乡镇级医疗卫生设施带有网络连接和网络服务器。 在乌帕齐拉的 482 个医疗卫生中心和地区医院中，建立起了移动医疗服务，有值班医生通过移动电话随时为患者提供咨询服务。 政府运作的远程医疗服务从 2014 年开始发起，43 个设施齐全的医疗卫生中心正在运营中。 这些医疗卫生中心安排职业医生访问乡村和偏远社区的诊所和联合保健中心，为患者提供医疗咨询。

**经验教训:**

尽管进行了早期试点干预以及有成功的实施，但是孟加拉国采用电子医疗战略的进展仍然缓慢。 由于缺少医疗卫生信息技术的通用标准，因此不同数据库之间的数据管理和分享存在困难。 有限的网络带宽和高成本的基础设施、软件设施发展是应用这些技术的障碍。

## Резюме

**Проблема:**

Инфраструктура единой информационной системы в сфере здравоохранения в Бангладеш еще только начинает развиваться, что совершенно необходимо для разработки и планирования направляющих принципов в этой области.

**Подход:**

В первых пробных программах телемедицины и системы электронного здравоохранения отсутствовало координирование на национальном уровне. Однако в 2011 году были приняты национальные стратегические принципы системы электронного здравоохранения (eHealth).

**Местные условия:**

За последние годы ситуация с системой здравоохранения в Бангладеш значительно улучшилась. Однако эта страна по-прежнему сталкивается с проблемами общественного здравоохранения. Сюда входят ограниченность и отсутствие равного доступа к медицинским услугам, а также недостаток соответствующих ресурсов на количество населения.

**Осуществленные перемены:**

В 2008 году в Бангладеш была запущена служба eHealth. Благодаря этому произошла компьютеризация медицинских учреждений на уровне районов, было обеспечено подключение к Интернету, созданы интернет-серверы и службы mHealth для организации связи с медицинскими работниками. Учреждениям здравоохранения районного уровня предоставили подключение к Интернету и серверам. В 482 больничных комплексах *упазилы* и районных больницах была создана служба mHealth. В рамках данной службы дежурный врач находится в режиме круглосуточного доступа для пациентов и может консультировать их при помощи мобильного телефона. Также была запущена правительственная служба телемедицины, и к 2014 году в Бангладеш работали уже 43 полностью оснащенных центра. Эти центры обеспечивают медицинские консультации квалифицированных врачей для пациентов, приходящих в сельские больницы, медицинские учреждения, расположенные в отдаленных регионах, а также для пациентов профсоюзных медицинских центров.

**Выводы:**

Несмотря на успешный запуск пробных проектов, стратегии системы электронного здравоохранения внедряются в Бангладеш медленно. В сфере здравоохранения в Бангладеш отсутствуют общепринятые стандарты информационных технологий. Это приводит к затруднению в управлении данными и обмене данными из разных баз. Ограниченная пропускная способность сети Интернет, дороговизна инфраструктуры и разработки программного обеспечения препятствуют широкому распространению таких технологий.

## Introduction

Despite substantial improvements in health in recent years, Bangladesh faces several challenges, including limited and inequitable access to health services, lack of adequate resources to meet the demands of the population and an increasing burden of noncommunicable diseases.^1^^,^^2^ Information and communications technologies – such as health information systems, mobile devices to support health systems (mHealth) and telemedicine services – can contribute to the improvement of health systems in developing countries.^3^ Here we describe the implementation of an eHealth policy in Bangladesh.

## Approach

A key aspect of the eHealth policy is the development of an integrated health information system, which includes a health management information system and an integrated human resource information system. A computerized health management information system provides tailored health services to stakeholders^4^ and a human resource information system integrates health workforce data from a range of sources such as ministries, agencies and health sector organizations. All information is stored in such a way that it can be easily found by users in different locations and in a form that is suited to their needs. The integrated health information system should meet international standards – such as ISO/TC 215 for health informatics – and provide access to all digital databases. The completed system combines individual health records of all citizens, registries of organizations, the hospital information system and health workforce data.^5^

## Relevant changes

Between 1999 and 2005, several telemedicine initiatives were initiated in Bangladesh, mainly to support rural doctors with expert opinions.^6^ In 2006 a mobile phone-based call centre was launched for subscribers.^4^ In 2008, eHealth services were introduced, including computerization of health facilities at sub-district levels, internet connections, internet servers and an mHealth service for communicating with health-care providers. The implementing authority, the Directorate General of Health Services, established a data centre equipped with modern servers, a backup safety system, firewalls, virtual machine software and information security systems to protect the safety of patient records.

The mHealth service is provided by 482 *upazila* health complexes and district hospitals. The *upazila* sub-district health centres have 50–100 bed capacity with an operating theatre and junior specialists. A doctor is available 24 hours a day to provide consultations by mobile phone.^5^ Subsequently, all community clinics and union health centres had internet connections installed and laptop computers provided by the Directorate General of Health Services. Several training workshops, which included lectures and demonstrations over several days, were organized by the ministry at district level. Selected health workers were given hand-held tablet devices.^5^

With recommendations from development partners and the World Health Organization (WHO), the Government of Bangladesh implemented a national eHealth policy in 2011.^7^^,^^8^ In July 2011, the Directorate General of Health Services inaugurated the telemedicine service. By 2014, a total of 43 fully equipped government-operated telemedicine centres were in service.^5^ These centres provide medical consultations via the internet by qualified physicians to patients visiting rural and remote community clinics and union health centres. The government also introduced a short message service (SMS) complaint–suggestion box to improve the accountability and transparency of public hospitals in Bangladesh. In about 800 public hospitals, a display board is mounted which describes how to send complaints about the quality of services or suggestions for service improvement to a mobile phone number. The government installed remote biometric time attendance machines in all *upazila* and district hospitals and in some tertiary hospitals to improve office attendance of staff. These low-cost machines can track attendance from the central office using locally-developed software.^5^

In 2014, 98 million individual electronic health records were generated from rural areas in Bangladesh, which will contribute to the population register for lifetime shared health records. The government has initiated partnership programmes with development and private organizations for implementing different eHealth services. For example, the Mobile Alliance for Maternal Action project by the government and D.Net, a development organization, provides information for pregnant women as well as advice for new mothers on how to care for their newborn infants and children. In collaboration with the Johns Hopkins Bloomberg School of Public Health, the mCare and mTikka projects track antenatal care and childhood immunizations.^5^

## Lessons learnt

The integration of information and communications technologies in the health system of Bangladesh faces several challenges and constraints, such as defining the services and standards across different organizations, the financial viability of the initiatives and the availability of technical staff.

There is a lack of common standards on health information and communications technologies and software, leading to difficulties in data management and sharing among different databases. Low internet connection speeds are a limitation in many areas. The high costs of infrastructure and integrated software development are also barriers to adoption of these technologies.

The private sector has fallen behind in the introduction of information and communications technologies and medical records from the private sector are not integrated with the national health database. A few large private hospitals have introduced eHealth and medical record systems. The government has plans to integrate data from the private sector and hopefully the large hospitals will soon join the system. However, organizing national representation of private sector organizations is challenging task. Box 1 summarizes the main lessons learnt.

Box 1 **Summary of main lessons learnt**

- Despite successful implementation of health information and communications technologies in Bangladesh, challenges still exist – such as technical problems, definition of services and standards across organizations and financial viability.
- Common standards for health information and communications technologies are needed to facilitate data management and sharing among different databases.
- The private sector was not included in implementation of the national eHealth programme and therefore medical records from this sector are not yet integrated with the national health database.

Despite early pilot interventions and successful implementation of several small-scale health projects using information technology, the progress in adopting eHealth strategies in Bangladesh has been rather slow and lacks robust data on effectiveness and cost–effectiveness which can provide evidence for scaling up to the national level. The information gathered in the health information system is starting to be evaluated. A recent study assessing the potential of an mHealth intervention for diabetes^9^ showed that mobile phone messages could be used to support the management of diabetes.^10^ The increasing popularity of eHealth services in developing countries can be explained by the rapid increase in mobile phone ownership and limited access to traditional health care and providers. eHealth promises a future where patients are more empowered with respect to their own health, community health workers use health diagnostic devices to monitor patients, to link with medical professionals and to track individuals.

These new methods of information sharing and delivery of services have the potential to improve the health of the population as they are low-cost and are readily accepted by users and service providers.^11^ However, development of an integrated health information system is a complex and costly process. Clinicians, managers, policy-makers and researchers need to be better informed about eHealth systems, so that the potential of new technology can be realized. Innovative information and communications technologies for health can strengthen health systems by providing services to underserved people in resource-poor settings, helping to achieve universal health coverage in Bangladesh as well as in other developing countries.
